# Centenarian SIRT6 variants elevate SIRT6 protein and enhance cellular senescence resistance

**DOI:** 10.21203/rs.3.rs-9997679/v1

**Published:** 2026-06-19

**Authors:** Yousin Suh, Jiping Yang, Xifan Wang, HyeRim Han, Yizhou Zhu, Lei Zhang, Haiqi Chen, Paul Robbins

**Affiliations:** Columbia University; Columbia University Medical Center; Columbia University Medical Center; Columbia University Irving Medical Centre; Columbia University Medical Center; University of Georgia; Columbia University Medical Center; University of Minnesota

## Abstract

Centenarians represent a natural model of delayed human aging, offering a unique opportunity to uncover genetic mechanisms that promote longevity. However, the functional consequences of the genetics variants carried by these long-lived individuals remain poorly characterized in physiologically relevant systems. Here, we introduced two linked missense variants in *SIRT6* enriched in Ashkenazi Jewish centenarians into the endogenous *SIRT6* locus of human embryonic stem cells and differentiated them into somatic lineages to define their effects in a native genomic context. We revealed that centenarian variants elevated endogenous SIRT6 protein abundance through weakened interaction with vimentin, and altered endogenous SIRT6 enzymatic activities, including enhanced mono-ADP-ribosyltransferase activity and reduced deacetylase activity. Functionally, these variants delayed replicative senescence and conferred resistance to progerin-induced stress, accompanied by preservation of DNA repair gene expression programs and suppression of transposable element derepression. Guided by these findings, we evaluated the translational potential of both genetic and pharmacological interventions, demonstrating that adeno-associated virus (AAV)-mediated delivery of centenarian SIRT6 or pharmacological activation of SIRT6 using fucoidan from *Fucus vesiculosus* (Fucoidan-FV) partially attenuated aging-associated molecular defects, including genome instability and LINE1 derepression, in progeria fibroblasts. Together, these findings demonstrate that centenarian variants exert multifaceted effects on SIRT6 function to enhance cellular stress resistance, and providing a framework for translating genetic discoveries from long-lived individuals into mechanistic insight and potential gerotherapeutic strategies for healthy aging.

## Introduction

Sirtuin 6 (SIRT6) is a nicotinamide adenine dinucleotide (NAD^+^)-dependent deacetylase and mono-ADP-ribosyltransferase (mADPr) that plays pivotal roles in DNA damage repair, chromatin stability, metabolic regulation, and stress responses^1–4^. SIRT6 overexpression extends lifespan and healthspan in mice^5,6^ and its double-strand break repair activity correlates with lifespan across species^7^, firmly establishing *SIRT6* as a longevity assurance gene.

Centenarians, who live not only longer but also show resistance and resilience to age-related diseases, represent a natural model of human longevity^8–10^. Genetic studies in exceptionally long-lived individuals have identified rare variants associated with extended lifespan^11–20^, including two linked missense variants in *SIRT6* (rs201141490-N308K and rs183444295-A313S) that are enriched in Ashkenazi Jewish centenarians^21^. We previously showed that the resulting centenarian SIRT6 protein (CentSIRT6) exhibits reduced deacetylase activity but enhanced mADPr activity, and augments DNA double-strand break repair and cancer cell killing in overexpression systems^21^. These protein-centric studies provided important insight into altered catalytic activities of CentSIRT6 but relied on ectopic expression in immortalized cell lines, a setting that does not preserve endogenous regulation of SIRT6 abundance and activity and therefore limits evaluation of variant effects on endogenous SIRT6 and downstream cellular phenotypes.

To overcome these limitations and define how centenarian *SIRT6* variants act in a native human genomic context, we generated knock-in human embryonic stem cells (hESCs) carrying the linked centenarian *SIRT6* variants and differentiated them into somatic lineages, focusing on human mesenchymal stromal cells (hMSCs) as a well-established model of cellular aging. This endogenous system allowed us to dissect how the variants influence SIRT6 regulation and activity and to assess their impact on cellular responses to replicative and stress-induced senescence using progerin, a causal factor of Hutchinson–Gilford progeria syndrome (HGPS). We further leveraged mechanistic insights from this model to test whether genetic or pharmacological modulation of SIRT6 can ameliorate molecular defects in HGPS fibroblasts. Collectively, these findings illuminate how rare centenarian variants modulate SIRT6 function and cellular resistance, expanding our understanding of the genetic and molecular basis of human longevity and providing avenues to translate genetic discoveries from long-lived individuals into gerotherapeutic strategies for healthy aging.

## Results

### Centenarian SIRT6 variants elevate endogenous SIRT6 protein levels

**Figure 1:**
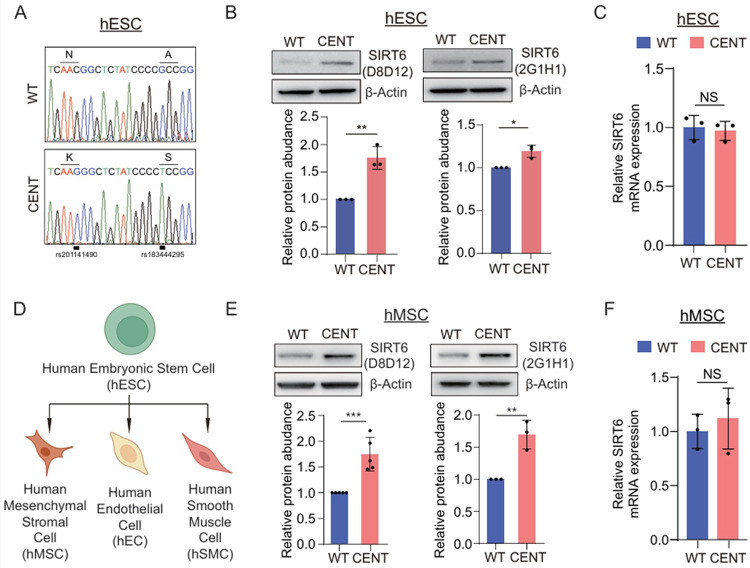
Elevated SIRT6 protein levels in hESCs and hESC-derived hMSCs carrying centenarian *SIRT6* variants. **(A)** Sanger sequencing confirming the knock-in of centenarian *SIRT6* variants in CENT hESCs. **(B)**SIRT6 protein levels in WT and CENT hESCs measured using two different SIRT6 antibodies (Clone ID: D8D12 and 2G1H1); Protein levels were normalized to β-Actin; data were represented as mean ± SD; n = 3; *, p < 0.05; **, p < 0.01. **(C)** SIRT6 mRNA levels in WT and CENT hESCs, gene expression was normalized to 18s rRNA; data were represented as mean ± SD; n = 3; NS, not significant. **(D)** Schematic showing the hESC-differentiated somatic cell types used in subsequent analyses. **(E)** SIRT6 protein levels in WT and CENT hMSCs; Protein levels were normalized to β-Actin; data were represented as mean ± SD; n = 3; **, p < 0.01; *** p < 0.001. **(F)** SIRT6 mRNA levels in WT and CENT hMSCs, gene expression was normalized to 18s rRNA; data were represented as mean ± SD; n = 3; NS, not significant.

To investigate the impact of centenarian *SIRT6* variants in an endogenous genomic context, we introduced the centenarian variants (rs183444295 and rs201141490) into hESCs (wild-type, WT) using CRISPR/Cas9-mediated gene editing (Fig. 1A). Successfully edited hESCs (centenarian, CENT) maintained pluripotency marker OCT4 expression, comparable to their isogenic controls **(Fig. S1A)**. Unexpectedly, CENT hESCs showed significantly elevated SIRT6 protein levels despite unchanged mRNA levels (Fig. 1B–1C). To assess whether this effect was cell-type specific, we differentiated hESCs into human mesenchymal stromal cells (hMSCs), human endothelial cells (hECs) and human smooth muscle cells (hSMCs) (Fig. 1D). Each lineage expressed its respective markers **(Fig. S1B-S1D)**, confirming lineage-committed differentiation. Notably, all three CENT hESC-derived lineages showed consistently elevated SIRT6 protein levels as confirmed using two antibodies recognizing distinct SIRT6 epitopes (Fig. 1E and S1F-G) but unchanged mRNA levels (Fig. 1F and S1E). Given that hMSC is a well-established cellular model for aging research^22–26^, we focused subsequent functional analyses on this cell type.

### Elevated SIRT6 is not caused by increased nuclear localization

**Figure 2:**
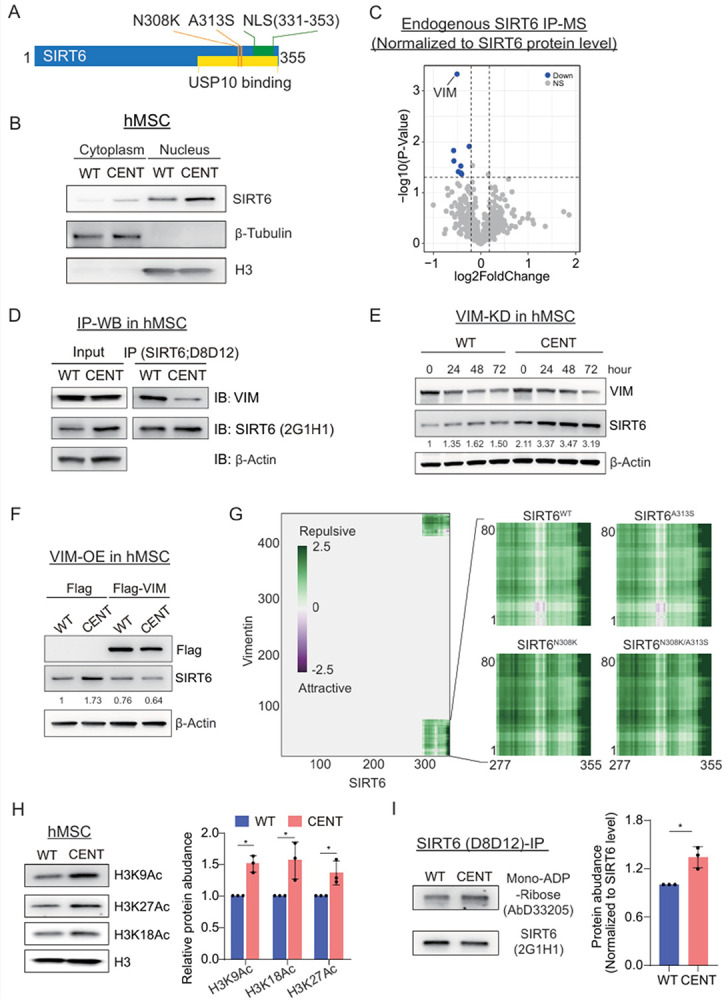
Impact of centenarian *SIRT6* variants on SIRT6 protein function in a cellular context **(A)** Schematic representation of the known domains in the SIRT6 protein. The green bar denotes the nuclear localization signal (NLS), orange lines indicate the positions of centenarian *SIRT6*mutations, and the yellow bar marks the C-terminal region required for USP10 binding. **(B)** SIRT6 protein levels in cytoplasmic and nuclear fractions of WT and CENT hMSCs. **(C)** Volcano plot showing differentially interacting proteins between WT SIRT6 and CentSIRT6 in hMSCs, identified by endogenous SIRT6 IP-MS. Protein abundances were normalized to SIRT6 levels in each pull-down sample. **(D)** Endogenous SIRT6 was immunoprecipitated from WT and CENT hMSC lysates using anti-SIRT6 antibody (D8D12). IP samples and input lysates were analyzed by western blotting with antibodies against SIRT6 (2G1H1) and vimentin (VIM). Input represents whole-cell lysate prior to immunoprecipitation. **(E)** Protein levels of vimentin and SIRT6 in WT and CENT hMSCs transfected with siRNAs targeting vimentin. Protein levels were normalized to β-Actin. **(F)** Protein levels of Flag-VIM and SIRT6 in WT and CENT hMSCs transduced with lentiviruses expressing Flag or Flag-VIM. Protein levels were normalized to β-Actin. **(G)** Predicted interaction between the IDRs of vimentin and the C-terminal IDR of SIRT6 using FINCHES. The right panel showing the effects of different SIRT6 mutations on SIRT6-vimentin interaction. **(H)** Protein levels of H3K9Ac, H3K27Ac and H3K18Ac in WT and CENT hMSCs. Protein levels were normalized to H3; data were represented as mean ± SD; n = 3; *, p < 0.05. **(I)** Relative proportion of mADP-ribosylated SIRT6 protein in hMSCs. Endogenous SIRT6 was immunoprecipitated from WT and CENT hMSC lysates using anti-SIRT6 antibody (D8D12). IP samples were analyzed by western blotting with antibodies against SIRT6 (2G1H1) and mono-ADP-Ribose (AbD33205). Protein levels were normalized to SIRT6; data were represented as mean ± SD; n = 3; *, p < 0.05.

Next, we investigated how the centenarian variants elevate SIRT6 protein levels. Since these centenarian mutations (N308K and A313S) are located proximal to the C-terminal nuclear localization signal (NLS; amino acids 331–353) (Fig. 2A), raising the possibility of altered nuclear import, we examined their effect on subcellular distribution. However, subcellular fractionation revealed elevated SIRT6 in both cytoplasmic and nuclear compartments of CENT hMSCs (Fig. 2B), ruling out enhanced nuclear import.

### Elevated SIRT6 is independent of its stabilizer USP10

SIRT6 stability is regulated by the ubiquitin-proteasome system through interactions with E3 ubiquitin ligases^27,28^ and deubiquitinating enzymes (DUBs)^29^. Among its established interactors, the DUB USP10 stabilizes SIRT6 by binding its C-terminal region^29^ - the region harboring the centenarian mutations (Fig. 2A). To test whether the elevated SIRT6 is mediated by USP10, we performed USP10 knockdown using siRNAs in both WT and CENT hMSCs. While USP10 knockdown reduced SIRT6 protein levels in WT hMSCs, SIRT6 protein levels in CENT hMSCs remained unchanged **(Fig. S2A)**, indicating that the increased SIRT6 levels in CENT hMSCs are independent of USP10.

### Vimentin mediates the increased SIRT6 protein

To identify mediators contributing to elevated SIRT6, we performed endogenous SIRT6 immunoprecipitation (IP) in WT and CENT hMSCs followed by tandem mass tag (TMT) - labeled mass spectrometry (MS). Strikingly, the most significantly altered SIRT6-interacting partner was vimentin (VIM), an intermediate filament protein (Fig. 2C **and Table. S1)**. Endogenous CentSIRT6 protein exhibited weaker interaction with vimentin compared to WT-SIRT6 (Fig. 2D). Moreover, vimentin knockdown by siRNAs increased SIRT6 protein levels in both WT and CENT hMSCs (Fig. 2E), whereas vimentin overexpression reduced SIRT6 levels in CENT hMSCs to those levels in WT hMSCs (Fig. 2F). These results demonstrate that vimentin mediates the increased SIRT6 in CENT hMSCs.

Given that the centenarian mutations reside within intrinsically disordered regions (IDRs)^2,30^, which are known to mediate protein–protein interactions^31^, we hypothesized that SIRT6-vimentin association is IDR-dependent. AlphaFold^32^ predictions revealed that SIRT6 harbors a C-terminal IDR (amino acids 298–355) and vimentin contains two IDRs at N-terminal (amino acids 1–89) and C-terminal (amino acids 409–422) regions **(Fig. S2B)**. To assess the binding potential between their IDRs, we employed FINCHES^33^, a sequence-based tool for predicting chemical-specific intermolecular interactions driven by disordered regions. The analysis revealed that SIRT6's C-terminal IDR encompassing the centenarian mutations showed potential to bind both the N-terminal and C-terminal IDRs of vimentin (Fig. 2G); however, the introduction of the two centenarian mutations in SIRT6 reduced the attraction to the N-terminal IDRs of vimentin (Fig. 2G), consistent with our experimental findings. Crucially, this weakened interaction was driven by the N308K mutation (Fig. 2G) rather than A313S which primarily enhances mADPr activity as we previously reported^21^. This indicates that N308K is uniquely responsible for the reduced SIRT6-vimentin interaction and the consequent increase in SIRT6 protein levels.

### Centenarian SIRT6 variants alter enzymatic activities in a cellular context

Given that CentSIRT6 protein displays weaker deacetylase activity but stronger mADPr activity in biochemical assays^21^, we assessed these activities in a cellular context using isogenic hMSCs. Despite having higher SIRT6 protein levels, CENT hMSCs showed increased histone acetylation at H3K9, H3K18, and H3K27 compared to WT hMSCs (Fig. 2H), confirming the reduced deacetylase activity of CentSIRT6. Additionally, we observed an increased proportion of mADP-ribosylated SIRT6 protein in CENT hMSCs (Fig. 2I), indicating enhanced mADPr activity of CentSIRT6. These results reveal that centenarian SIRT6 variants alter enzymatic activities in a cellular context, in line with previous biochemical findings^21^.

### Centenarian SIRT6 variants moderately alter the transcriptome in young hMSCs

**Figure 3:**
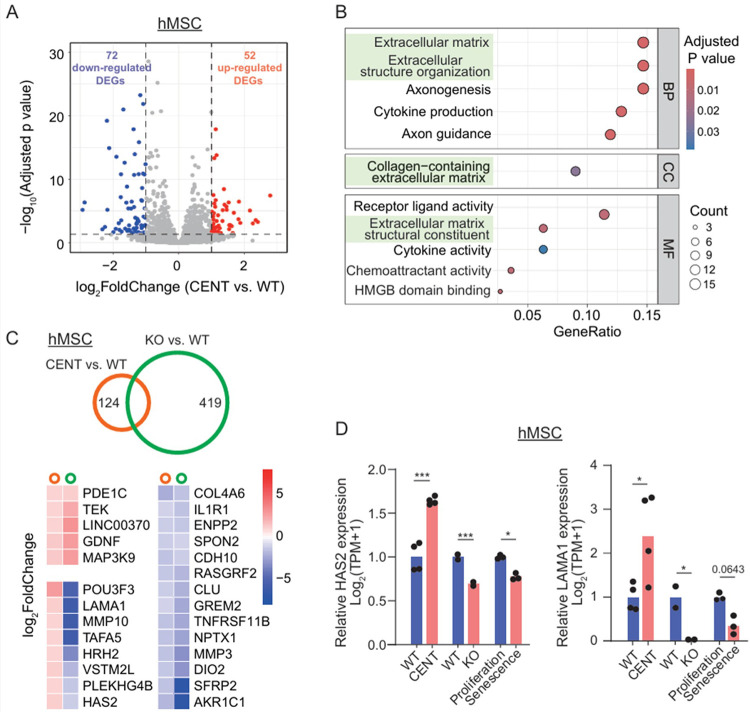
Moderate transcriptomic changes between WT and CENT hMSCs at early passage. **(A)** Volcano plot showing the differentially expressed genes (DEGs) identified by RNA-seq of WT and CENT hMSCs at early passage (P3). **(B)** Gene ontology (GO) analysis of DEGs between WT and CENT hMSCs. Extracellular matrix (ECM)-associated GO terms were highlighted in green. **(C)** Venn diagram showing the overlap of DEGs identified in CENT vs. WT hMSCs and *SIRT6*-KO vs. WT hMSCs. The lower panel presents the overlapping genes along with their expression changes in both comparisons. **(D)** RNA-seq expression profiles of ECM-related genes *HAS2* and *LAMA1* in CENT vs. WT hMSCs, *SIRT6*-KO vs. WT hMSCs, and proliferating vs. senescent hMSCs. Asterisks indicate statistical significance based on DESeq2 analysis using adjusted p-values; *, p < 0.05; ***, p < 0.001. Normalized expression values (TPM: Transcripts Per Million) were used for plotting; data were represented as mean.

To investigate the molecular changes caused by centenarian *SIRT6* variants, we performed transcriptome analysis on WT and CENT hMSCs at early passage. Differentially expressed gene (DEG) analysis demonstrated that replacement of two nucleotides altered the hMSC transcriptome, revealing 52 down-regulated and 72 up-regulated DEGs (|Fold Change| > 2, adjusted p value < 0.05) in CENT versus WT hMSCs (Fig. 3A **and Table. S2)**. Gene ontology analysis showed that these DEGs were significantly enriched in extracellular matrix (ECM) (Fig. 3B).

To identify genes potentially regulated directly by SIRT6, we integrated transcriptome data from *SIRT6*-knockout hMSCs^34^, which revealed 419 DEGs relative to SIRT6-WT hMSCs (Fig. 3C). Among these, 27 genes overlapped with those affected by the centenarian variants, showing both concordant and opposite expression changes (Fig. 3C), suggesting that the effects of centenarian *SIRT6* variants are more complex than simple upregulation or downregulation of SIRT6. Notably, the ECM genes *HAS2* and *LAMA1*, which were downregulated in *SIRT6*-knockout hMSCs and during hMSC senescence, were significantly upregulated in CENT hMSCs (Fig. 3D). Interestingly, *HAS2*, encoding hyaluronan synthase 2, is an ECM component that has been linked to improved healthspan^35^.

We also examined genes in pathways previously linked to SIRT6 including glycolysis^36,37^, lipogenesis^37^, autophagy^38,39^, oxidative stress^34^ and cancer^2^, but most of these genes showed no significant differences between WT and CENT hMSCs **(Fig. S3A-S3E)**. Taken together, the *SIRT6* variants exerted only moderate effects on the transcriptome in young hMSCs, which remained largely homeostatic and minimally stressed.

### Centenarian SIRT6 variants delay replicative senescence

Given that SIRT6 is highly dynamic and stress-responsive^40^, we next investigated the centenarian variants in hMSCs under stress conditions, examining their effects on cellular aging through replicative senescence induced by serial passaging **(Fig. S4A)**. At early passages, WT and CENT hMSCs showed similar proliferation and senescence-associated β-galactosidase (SA-β-gal) activity **(Fig. S4B-S4C)**. However, at late passages, CENT hMSCs exhibited a higher proportion of Ki67-positive cells and fewer SA-β-gal-positive cells **(Fig. S4B-S4C)**, demonstrating delayed senescence compared to WT hMSCs.

### Centenarian SIRT6 variants confer resistance to progerin-induced senescence

**Figure 4:**
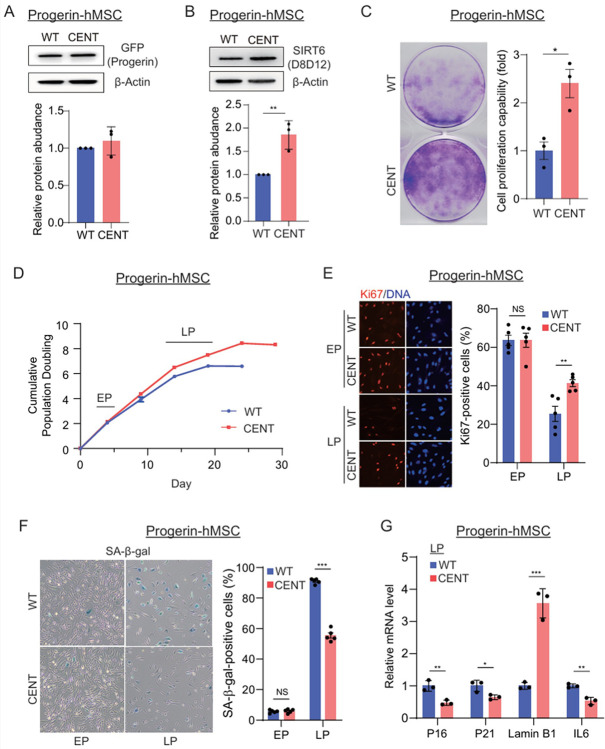
Centenarian *SIRT6* variants confer resistance to progerin-induced stress. **(A)** Progerin protein levels in WT and CENT hMSCs transduced with GFP-progerin lentiviruses (Progerin-hMSCs). Protein levels were normalized to β-Actin; data were represented as mean ± SD; n = 3; NS, not significant. **(B)** SIRT6 protein levels in WT and CENT Progerin-hMSCs. Protein levels were normalized to β-Actin; **, p < 0.01. **(C)** Clonal formation analysis of WT and CENT Progerin-hMSCs; data were represented as mean ± SD; n = 3. *, p < 0.05. **(D)** Growth curves of WT and CENT Progerin-hMSCs. EP (early passage, 2 passages post-Progerin overexpression and sorting); LP (late passage, 4–5 passages post-Progerin overexpression and sorting), data were represented as mean ± SD, n = 2. **(E)**Immunofluorescence staining of Ki67 in WT and CENT Progerin-hMSCs at EP and LP. Quantification based on 5 independent images capturing over 500 nuclei; data were represented as mean ± SD; NS, not significant; **, p < 0.01. **(F)** SA-β-gal staining in WT and CENT Progerin-hMSCs at EP and LP. Quantification based on 5 independent images capturing over 500 nuclei; data were represented as mean ± SD; NS, not significant; ***, p < 0.001. **(G)** Relative mRNA levels of P16, P21, Lamin B1 and IL6 in WT and CENT Progerin-hMSCs at LP; gene expression was normalized to 18s rRNA; data were represented as mean ± SD; n = 3, *, p < 0.05; **, p < 0.01; ***, p < 0.001.

Since SIRT6 maintains chromatin organization and DNA repair, cellular processes that are disrupted by progerin^41^, we further evaluated the impact of the centenarian *SIRT6* variants in a more physiologically relevant disease context: Hutchinson-Gilford Progeria Syndrome (HGPS). HGPS is a premature aging disorder caused by progerin, a toxic isoform of Lamin A. We generated WT and CENT hMSCs stably expressing GFP-progerin (Progerin-hMSCs)^42^. Both lines showed comparable progerin levels (Fig. 4A), with CENT Progerin-hMSCs retaining elevated SIRT6 protein levels (Fig. 4B). Colony formation assays revealed enhanced proliferative potential of CENT Progerin-hMSCs (Fig. 4C). Moreover, centenarian variants strongly ameliorated multiple cellular aging phenotypes (Fig. 4D), as evidenced by a higher percentage of Ki67-positive cells (Fig. 4E), reduced SA-β-gal activity (Fig. 4F), decreased expression of aging markers P16 and P21, reduced expression of the senescence-associated secretory factor (SASP) gene IL6, and increased levels of Lamin B1 (Fig. 4G).

### Centenarian SIRT6 variants maintain genome stability under progerin-induced stress

**Figure 5:**
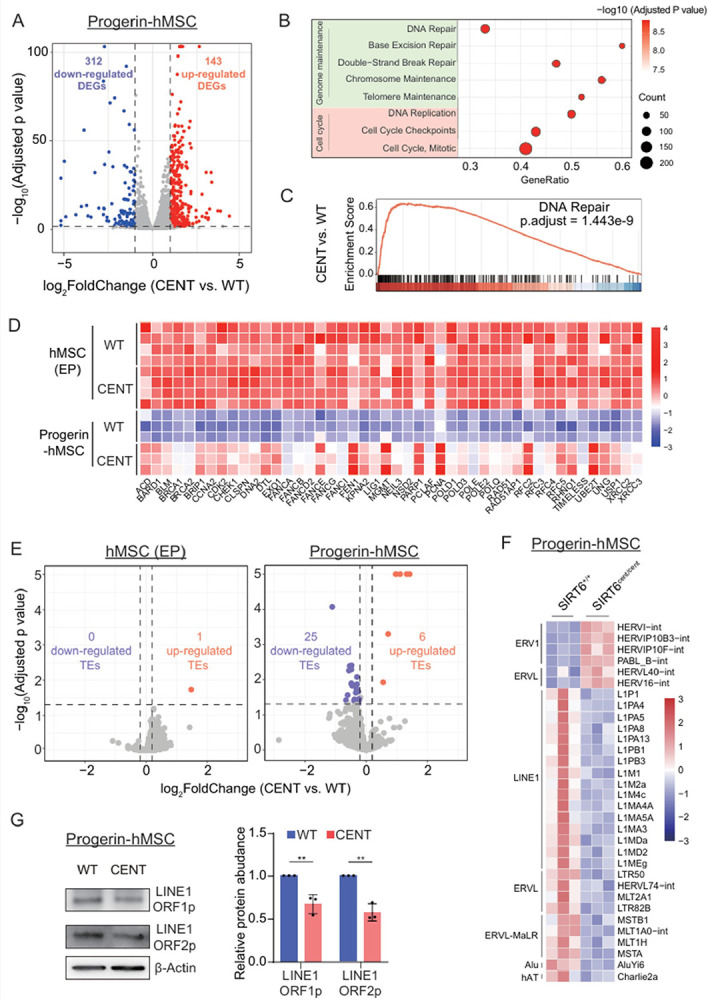
Centenarian *SIRT6* variants maintain genome stability under progerin-induced stress. **(A)** Volcano plot showing the differentially expressed genes (DEGs) identified by RNA-seq of WT and CENT Progerin-hMSCs at 3 passages post-Progerin overexpression and sorting. **(B)** Gene set enrichment analysis (GSEA) revealing pathways altered by centenarian *SIRT6*variants were enriched in cell cycle regulation and genome maintenance. **(C)**GSEA enrichment plot showing downregulation of genes involved in DNA repair pathways in CENT Progerin-hMSCs compared to WT Progerin-hMSCs. **(D)**Heatmap showing the expression of DNA repair genes in hMSCs with or without Progerin-induced stress. **(E)** Volcano plot showing the differentially expressed transposable elements (DETEs) identified in EP hMSCs (Left) and Progerin-hMSCs (Right). **(F)** Heatmap displaying the expression levels of DETEs in Progerin-hMSCs. **(G)** Protein levels of LINE1-ORF1p and LINE1-ORF2 in WT and CENT Progerin-hMSCs. Protein levels were normalized to β-Actin; data were represented as mean ± SD; n = 3; **, p < 0.01.

Next, we investigated the molecular mechanisms by which the centenarian *SIRT6* variants confer protection against progerin. Transcriptome analysis of WT and CENT Progerin-hMSCs identified 312 down-regulated and 143 up-regulated DEGs (|Fold Change| > 2, adjusted p value < 0.05) in CENT versus WT hMSCs (Fig. 5A **and Table S3)**. The increased number of DEGs under progerin-induced stress, relative to early passage cells, highlights SIRT6’s dynamic response to stress. Strikingly, pathways related to cell cycle regulation and genome stability including not only DNA damage repair such as double-strand break (DSB) and base excision repair (BER) but also higher-order structures such as chromosome and telomere maintenance were significantly altered by the centenarian variants (Fig. 5B–5C). At early passage without progerin, DNA repair genes showed comparable expression in WT and CENT hMSCs (Fig. 5D). With progerin expression, the expression of these genes was markedly decreased in WT hMSCs but largely preserved in CENT hMSCs (Fig. 5D), suggesting that the centenarian *SIRT6* variants primarily ameliorate progerin-induced stress through preserving genome stability.

### Centenarian SIRT6 variants suppress progerin-Induced TE derepression

Progerin triggers heterochromatin loss^43^, leading to the derepression of transposable elements (TEs) such as LINE1^44^ and HERVs^45^. SIRT6 maintains TE silencing, but this fails in senescent cells or aged tissues, allowing TEs to become transcriptionally active and contribute to genomic instability^46,47^. We therefore examined whether centenarian *SIRT6* variants could mitigate progerin-induced TE reactivation. We found minimal changes in hMSCs at early passage (Fig. 5E **and Table. S4)**, but upon progerin-induced stress, CENT hMSCs showed better-maintained suppression (Fig. 5E **and Table. S5)**, particularly of LINE1 elements (Fig. 5F), compared to WT hMSCs. We further confirmed reduced LINE1 ORF1p and ORF2p proteins (Fig. 5G), both translated from LINE1 RNAs, in Progerin-hMSCs carrying centenarian *SIRT6* variants. Additionally, the majority of non-LINE TEs also exhibited reduced expression in CENT hMSCs compared to WT hMSCs following progerin overexpression **(Fig. S5A)**, suggesting globally preserved genome integrity conferred by centenarian *SIRT6* variants.

### Centenarian SIRT6 variants-based genetic and pharmacological interventions attenuate molecular defects in progeria fibroblasts

**Figure 6:**
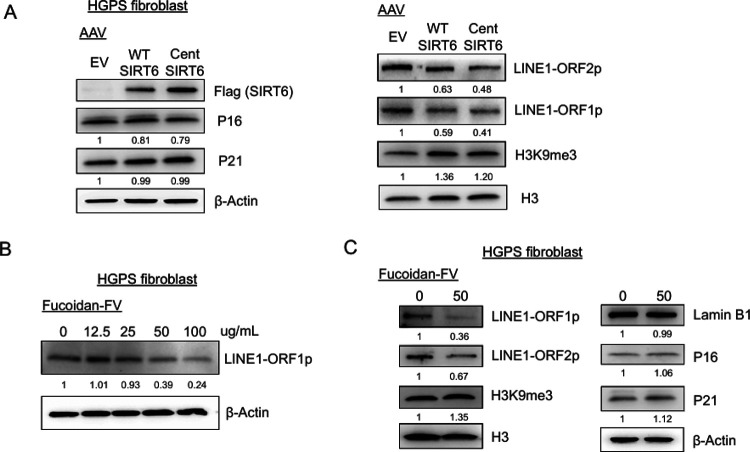
Treatment with SIRT6 activator Fucoidan-FV downregulates LINE1 in progeria patient cells **(A)** Protein levels of senescence markers in HGPS patient fibroblasts transduced with AAV viruses expressing empty vector (EV), WT-SIRT6 or CentSIRT6. P16 and P21 protein levels were normalized to normalized to β-Actin; LINE1-ORF1p, LINE1-ORF2p and H3K9me3 were normalized to H3. **(B)** LINE1-ORF1p protein levels in human progeria fibroblasts treated with Fucoidan-FV at different dosages (0–100 μg/mL) for 7 days. Protein levels were normalized to β-Actin. **(C)** Protein levels of senescence markers in HGPS patient fibroblasts treated with or without 50 μg/mL Fucoidan-FV. P16 and P21 protein levels were normalized to normalized to β-Actin; LINE1-ORF1p, LINE1-ORF2p and H3K9me3 were normalized to H3.

Guided by these mechanistic insights, we evaluated the therapeutic potential of centenarian SIRT6 in ameliorating premature aging phenotypes in HGPS patient-derived fibroblasts. Adeno-associated virus (AAV)-mediated delivery of SIRT6 reduced LINE1 ORF1p and ORF2p protein levels compared to empty vector controls, with CentSIRT6 exhibiting a more pronounced reduction (Fig. 6A). While SIRT6 overexpression trended toward counteracting senescence-associated molecular changes, the effects on SA-β-gal activity and proliferation were not obvious **(Fig. S6A-S6B)**.

To explore a pharmacological intervention mimicking the effects of centenarian *SIRT6* variants, we tested Fucoidan from *Fucus vesiculosus* (Fucoidan-FV), a SIRT6 activator that elevates SIRT6 levels and enhances mADPr activity^48,49^. Dose-dependent screening in progeria fibroblasts revealed that 50 and 100 μg/mL of Fucoidan-FV successfully reduced LINE1 ORF1p and ORF2p levels (Fig. 6B and 6C). Fucoidan-FV did not alter the proportion of SA-β-gal–positive or Ki67-positive cells **(Fig. S6C-S6D)**. Collectively, these results suggest that both centenarian *SIRT6* variants-guided genetic and pharmacological interventions can specifically mitigate molecular defects, particularly LINE1 reactivation, although their impact on broader senescence phenotypes is limited, likely due to the near-senescent state of the HGPS patient-derived cells.

## Discussion

Centenarians, who live not only longer but also healthier, represent an important model for understanding mechanisms that promote healthy aging^8,9,50^. Genetic studies have identified variants enriched in exceptionally long-lived individuals, yet how these variants act at cellular and molecular levels remains unclear^51,52^. In this study, we employed CRISPR-mediated knock-in of centenarian *SIRT6* variants in hESCs followed by lineage-committed cell differentiation to examine variant function in an endogenous genomic context. This strategy enabled interrogation of variant effects on protein regulation, enzymatic activity and cellular stress responses in human cells without the confounding effects of ectopic overexpression. This endogenous knock-in platform, combined with hESC-derived somatic lineages, provides a generalizable way to functionally characterize human longevity-associated variants in defined cellular contexts.

Our findings reveal that centenarian *SIRT6* variants exert multifaceted effects on SIRT6 biology. In addition to previously reported alterations in catalytic activity, we identify a distinct IDR-mediated mechanism regulating SIRT6 protein abundance through weakened interaction with vimentin. Vimentin, traditionally recognized as an intermediate filament protein providing structural support, has more recently been implicated in autophagy^53^ and vesicle trafficking^54,55^, underscoring its role in proteostasis. Notably, vimentin has been reported to undergo liquid–liquid phase separation (LLPS) to form droplets^30^, a process that depends on its IDR. Although the precise mechanism by which vimentin influences SIRT6 stability remains to be defined, our data support a model in which the N308K substitution selectively reduces SIRT6-vimentin interaction, leading to increased SIRT6 protein levels.

At the cellular level, centenarian *SIRT6* variants conferred resistance to both replicative and progerin-induced stress in hMSCs. Notably, transcriptomic differences between WT and CENT hMSCs were modest under basal conditions, suggesting that variant effects are context-dependent and become more pronounced during stress exposure. Under progerin-induced perturbation, centenarian variants were associated with preservation of gene expression programs related to genome maintenance and reduced derepression of transposable elements, particularly LINE1 elements. These observations are consistent with established roles for SIRT6 in DNA repair^56^ and transposon silencing^46,47^ and suggest that the centenarian variants preferentially support stress-responsive genome stability pathways rather than broadly reprogramming cellular transcriptional networks.

Our variant-to-function study highlights several potential translational implications. First, given that SIRT6 overexpression extends healthspan and lifespan in mice, systematic delivery of CentSIRT6 via gene delivery approaches, such as adeno-associated virus (AAV), could represent a promising gene therapy strategy to counteract aging-associated declines. Second, centenarian variants can be leveraged to genetically enhance stem cell function for cell therapy, a concept similar to recent studies introducing artificial mutations in the longevity gene *FOXO3* to generate senescence-resistant MSCs (SRCs) which counter aging in primates^57^. Unlike engineered mutations that may occur at sites also mutated in human cancers^57^, centenarian variants occur naturally in long-lived individuals who maintain extended healthspan for a century, suggesting that they may provide a safer starting point for genetic enhancement strategies. Third, our study provides a strategy to develop SIRT6 activators for geroprotection. Existing SIRT6 activators primarily enhance deacetylase activity^2^, whereas our findings highlight modulation of SIRT6 mADPr activity as a promising target.

The centenarian *SIRT6* variants confer multifaceted effects on SIRT6 protein. This complex gain-of-function illustrates why rare missense variants cannot be phenocopied by conventional gene overexpression or knockdown strategies commonly used for studying GWAS variants. Our variant knock-in and hESC differentiation paradigm partially addresses these variant-to-function challenges, primarily revealing molecular and cellular changes such as stress response and senescence. Nevertheless, tissue-specific consequences of centenarian *SIRT6* variants and their impact *in vivo* remain to be determined. Future studies should leverage human hESC-derived organoids to directly interrogate variant effects in physiologically relevant tissue contexts. Additionally, given that these variants are not conserved in rodents, humanized *SIRT6* mice or non-human primates represent optimal models to evaluate their impact on healthspan and lifespan *in vivo*.

In conclusion, this study provides a framework for functionally characterizing rare centenarian variants and translating genetic discoveries into mechanistic insight and potential therapeutic interventions. This functional genomics study on centenarian *SIRT6* variants demonstrates how naturally occurring variations in long-lived individuals can guide the development of strategies to improve healthspan and mitigate age-related declines.

## Methods and Materials

### Antibodies

Antibodies for western blotting (WB) and immunofluorescence (IF) staining were obtained from the following sources.

BD Bioscience: CD73-PE (561014), CD90-FITC (555595), CD105-APC (562408), CD31-FITC (555445), P16 (550834);

Santa Cruz Biotechnology: OCT4 (sc-5279), β-Actin (sc-47778), SIRT6 (2G1H1, sc-517196), GFP (sc-9996), β-Tubulin (sc-5274), VIM (sc-6260), Lamin A/C (sc-376248);

Millipore Sigma: H3K9Ac (07–352), SMA (A5228), Flag (F1804), LINE1-ORF1p (MABC1152);

Cell Signaling Technology: SIRT6 (D8D12, 12486), USP10 (5553), H3 (9715), P21 (2947);

Abcam: H3K18Ac (ab1191), H3K9me3 (ab8898), Lamin B1 (ab133741), LINE1-ORF2p (ab106004);

Active Motif: H3K27Ac (39034);

Thermo Fisher: Ki67-APC (17-5699-42);

Bio-Rad: Mono-ADP-Ribose (AbD33205).

For endogenous SIRT6 Immunoprecipitation (IP), SIRT6 (D8D12, 12486) antibodies were used in IP, SIRT6 (2G1H1, sc-517196), VIM (sc-6260) and Mono-ADP-Ribose (Bio-Rad, AbD33205) antibodies were used in immunoblotting (IB).

### Cell culture

H7 human embryonic stem cells (ESC) (WA07, WiCell) were maintained on Matrigel (BD Biosciences) in mTeSR Plus medium (STEMCELL Technology).

Mesenchymal stromal cells were culture in aMEM (Gibco) with 10% fetal bovine serum (FBS, Hyclone or GeminiBio), and 1 ng/mL bFGF (Thermo Fisher).

Endothelial cells were cultured in EGM-2 endothelial cell growth medium (Lonza CC-3162) supplemented with 10nM SB431542 (Selleck), 50 ng/mL VEGF (Peprotech) and 20ng/mL bFGF (Thermo Fisher).

Smooth muscle cells were cultured in N2B27 Medium supplemented with 2ng/mL Activin A (Thermo Fisher) and 2ug/mL Heparin (STEMCELL Technology).

Fibroblasts isolated from Hutchinson–Gilford Progeria Syndrome (HGPS) patients (Coriell, AG11513) were cultured in DMEM (Gibco) with 15% fetal bovine serum (FBS, Hyclone) in hypoxia incubator chamber (STEMCELL Technology) filled with Tri-Gas containing 5% CO2 and 5% O2 for maintenance and expansion. Fucoidan derived from *Fucus vesiculosus* (Fucoidan-FV) was purchased from Sigma-Aldrich (F8190) and kindly provided by Paul D. Robbins (University of Minnesota).

All cell culture media were supplemented with 1% penicillin/streptomycin (Gibco) and 50ug/mL Normocin (InvivoGen) to prevent cells from microbial contaminations. Cultured cells were routinely tested for mycoplasma contamination using Mycoplasma PCR Detection Kit (ABM). Unless otherwise specified, all cells were maintained at 37°C in a humidified atmosphere containing 5% CO2 and 20% O2.

### Variant editing at *SIRT6* locus

CRISPR/Cas9-mediated knock-in was performed using Alt-R CRISPR-Cas9 System (IDT) and HDR Donor Oligos. Cas9 nuclease, SIRT6 gRNA (GAATCTCCCACCCGGATCAA) targeting double variant site and single-strand DNA oligo donors (ssODNs) harboring centenarian *SIRT6* variants were ordered from IDT. To generate centenarian SIRT6 variants knock-in hESCs, 2´10^5 individualized hESCs were resuspended in 10 mL Resuspension Buffer R (Invitrogen) containing CRISPR ribonucleoproteins (Cas9 protein+gRNA) and ssODNs and were then electroporated using NEON Transfection System (Invitrogen). After electroporation, cells were seeded on Matrigel-coated plates in mTeSR plus with 1x RevitaCell Supplement (Gibco). After 48h expansion, cells were dissociated by Accutase and 10,000 cells were seeded on CytoSort^™^ Array (10,000 microwells, CELL Microsystem). Once cells were attached, microwells containing single colony were automatically picked and transferred to 96-well plate by CellRaft AIR System (CELL Microsystem). The expanded clones on 96-well plate were genotyped by TaqMan genotyping assay (rs201141490, rs183444295, Thermo Fisher) and further confirmed by sanger sequencing of PCR products amplified using primers AGCCTCACCTCTGGACAACACAGCAA and TCGTCAACCTGCAGCCCACCAAGCAC.

### Mesenchymal stromal cell differentiation

hMSCs were differentiated from hESCs as previously described with minor modification^58^. Briefly, embryoid bodies were first produced using AggreWell (STEMCELL Technology) and were then plated on Matrigel-coated plates in hMSC differentiation medium (aMEM (Gibco), 10% fetal bovine serum (GeminiBio), 1% penicillin/streptomycin (Gibco), 10 ng/mL bFGF (Thermo Fisher) and 5 ng/mL TGFβ (Thermo Fisher)) for around 10 days till fibroblast-like cells were confluent. These fibroblast-like cells were maintained in hMSC culture medium on Gelatin-coated 10 cm dishes for two passages and were further sorted (BD FACSAria II) to purify CD73/CD90/CD105 tri-positive hMSCs.

### Endothelial cell differentiation

Differentiation of hESCs into hECs was performed as previously described with minor modifications^59^. Briefly, hESCs were cultured in mTeSR1-Plus media (STEMCELL Technology) for one day and then in M1 medium, containing IWP2 (3 mM, Selleck), BMP4 (25 ng/ml, Peprotech), CHIR99021 (3 mM, Selleck) and bFGF (4 ng/ml, Thermo Fisher), for three days. The following day, M1 medium was removed and replaced with M2 medium with the addition of VEGF (50 ng/ml, Peprotech), bFGF (20 ng/ml, Thermo Fisher) and IL6 (10 ng/ml, Peprotech) to promote endothelial cell emergence for another three days. The differentiated adherent cells were harvested using TrypLE (GIBCO), labeled with CD144 (VECadherin) MicroBeads (130-097-857, Miltenyi Biotec), and separated by OctoMACS^™^ Separator (Miltenyi Biotec).

### Growth curve assay

Cell population doubling was determined as previously described^58^. Briefly, hMSCs were serially passaged and the number of cells was counted after dissociation. Population doubling per passage was calculated as log2 (number of cells harvested/number of cells seeded). Cumulative population doublings of the cells were calculated and plotted against culture days.

### Senescence-associated β-galactosidase (SA-β-gal) staining

The SA-β-gal staining of hMSCs was conducted as previously described^58^. Briefly, cells were washed with PBS and fixed at room temperature for 5 minutes using a fixation buffer containing 2% formaldehyde and 0.2% glutaraldehyde. After fixation, cells were washed with PBS and incubated overnight at 37 °C with freshly prepared staining solution (1 mg/mL X-gal, 40 mM citric acid/sodium phosphate pH 6.0, 5 mM potassium ferrocyanide, 5 mM potassium ferricyanide, 150 mM NaCl, 2 mM MgCl_2_). Images were acquired using the EVOS Cell Imaging System (Thermo Fisher). SA-β-gal-positive and total cell numbers were quantified using ImageJ, and the percentage of SA-β-gal-positive cells was calculated for statistical analysis.

### Immunoprecipitation

For endogenous co-immunoprecipitation assays, WT and CENT hMSCs were lysed in ice-cold IP buffer composed of 50 mM Tris-HCl (pH 7.5), 150 mM NaCl, 1% NP-40, and 5 mM EDTA, supplemented with 1× Protease and Phosphatase Inhibitor Cocktail (Roche). Lysates were cleared by centrifugation at 13,000 rpm for 15 minutes at 4 °C and further quantified using BCA Kit (Thermo Fisher). Equal amounts of lysates from WT and CENT hMSCs were pre-cleared with Pierce Protein A/G Plus Agarose (Thermo Fisher) for 2 hours at 4 °C with rotation. Agarose were separated by centrifugation at 13,000 rpm for 5 minutes at 4 °C. The supernatant was transferred to fresh tubes and incubated with the SIRT6 antibodies (D8D12, Cell Signaling Technology) overnight at 4 °C under constant rotation. The next day, Pierce Protein A/G Plus Agarose (Thermo Fisher) was added to bind antibodies for 2 hours. The beads were washed five times with ice-cold IP buffer and interacting proteins were eluted in Elution Buffer (100 mM Tris-HCl, pH 7.5, 1% SDS) using thermomixer at 65 °C for 10 minutes. Eluted samples were analyzed by western blotting or sent to Innomics for mass spec analysis.

### Mass spectrometry

TMT quantitation on SIRT6-IP samples was performed by Innomics. Samples were first assayed for protein content using the BCA method and then processed using the S-Trap MS sample prep device (PROTIFI) according to the manufacturer’s protocol. Briefly, samples were first reduced with dithiothreitol (DTT) and alkylated with iodoacetamide (IAM) and the resulting samples were digested in the Strap with Trypsin/Lys-C overnight. Digested samples were eluted and then dried by Speed-Vac. The dried samples were reconstituted with 100mM TEAB pH 8.5. All samples were added with their assigned TMT channels. The TMT-added samples were incubated at room temperature for 1 hour for labelling. About 1% of each TMT-labelled sample was aliquoted and mixed with 1% formic acid. TMT-labelled composite was loaded onto the nano LC-MS/MS system for Label Check. After the Label Check was passed, all samples were dried by Speed-Vac and reconstituted with 2% formic acid. The samples were combined into one mixture. The resulting mixture was further desalted using EVOLUTE^®^ EXPRESS ABN according to the manufacturer’s protocol (Biotage). The eluted samples were dried by SpeedVac, reconstituted with high-pH reversed-phase buffer A, and fractioned via offline HPLC fraction. Fraction samples were reconstituted with mobile phase A and loaded onto the nano LC-MS/MS system. About 1uL of each reconstituted sample was injected for analysis with TMT method. TMT quantification was performed with Proteome Discoverer 2.5 (Thermo Fisher). The quantification results were provided in Table. S1.

### Western blotting

Cells were lysed in RIPA buffer (Thermo Fisher) with protease inhibitor cocktail (Roche). Protein quantification was performed using a BCA Kit (Thermo Fisher). Protein lysate was subjected to SDS-PAGE and subsequently electrotransferred to a PVDF membrane (Bio-Rad) using wet transfer. Then primary antibodies and HRP conjugated secondary antibodies were incubated with the 5% milk blocked membrane. The imaging and quantification of target proteins was obtained by ChemiDoc MP imaging system (Bio-Rad).

### RT-qPCR

Total RNA was extracted using RNeasy Mini Plus Kit (Qiagen). Then One-step PrimeScript RT-PCR kit (Takara) was used to generate cDNA. RT-qPCR was performed with PowerUp SYBR Green Master Mix (Thermo Fisher) in QuantStudio 6 Pro Real-Time PCR System (Thermo Fisher). All primer sequences for qPCR are listed below:

SIRT6- Forward: 5′-CCCACGGAGTCTGGACCAT-3′, Reverse: 5′- CTCTGCCAGTTTGTCCCTG-3′;

P16- Forward: 5′-ATGGAGCCTTCGGCTGACT-3′, Reverse: 5′- GTAACTATTCGGTGCGTTGGG-3′;

P21- Forward: 5′-CGATGGAACTTCGACTTTGTCA-3′, Reverse: 5′- GCACAAGGGTACAAGACAGTG-3′;

Lamin B1- Forward: 5′-GTAAGCACTGATTTCCATGTCCA-3′, Reverse: 5′- GAAAAAGACAACTCTCGTCGCA-3′;

IL6- Forward: 5′- ACTCACCTCTTCAGAACGAATTG-3′, Reverse: 5′- CCATCTTTGGAAGGTTCAGGTTG-3′.

### Immunofluorescence staining

Cells were fixed in 4 % paraformaldehyde at room temperature (RT) for 15 min, permeabilized in 0.4% Triton X-100/PBS at RT for 10 min. After blocking with 10% donkey serum (Jackson ImmunoResearch Labs)/PBS for 1 h, cells were incubated with primary antibodies at 4 °C overnight and the corresponding secondary antibody (Invitrogen) at RT for 45 min. Nuclei were stained with Hoechst 33342 (Thermo Fisher 62249).

### siRNA transfection

siRNAs targeting human USP10 (sc-365828) and Vimentin (sc-373717) were purchased from Santa Cruz Biotechnology. siRNAs were transfected into hMSCs using Lipofectamine RNAiMAX Reagent (Thermo Fisher) according to the manufacturer's instructions.

### Plasmids

For flag-VIM overexpression in hMSCs, VIM CDS were amplified from cDNA of H7 ESC-derived hMSCs using primers (TGTCGTGACAAGTTTGTACAGCCACCATGGACTACAAAGACGATGACGACAAGTCCACCAGGTCCGTGTCCTCGTCCTCCTAC and TCGATTATCACCACTTTGTACATTATTCAAGGTCATCGTGATGCTGAGAAGT). Purified PCR products were cloned into pLV-Neo-EF1A backbone (VectorBuilder) using NEBuilder (NEB) according to the manufacturer's instructions. Control plasmid expressing Flag tag was constructed by the insertion of annealed Flag sequences (GTACAGCCACCATGGACTACAAAGACGATGACGACAAGTAGT and GTACACTACTTGTCGTCATCGTCTTTGTAGTCCATGGTGGCT) into pLV-Neo-EF1A backbone using Quick Ligase (NEB). Plasmid sequences were validated using whole plasmid sequencing service provided by Genewiz.

For GFP-progerin overexpression in hMSCs, GFP-progerin CDS were amplified from pBABE-puro-GFP-progerin plasmids (Addgene, 17663) and cloned into pLenti4 backbone.

For AAV-mediated WT-SIRT6 and CentSIRT6 overexpression, WT-SIRT6 CDS along with an N-terminal Flag tag was cloned into pAAV-CBh backbone (VectorBuilder) using NEBuilder (NEB) according to the manufacturer's instructions. pAAV-CBh-Flag-CentSIRT6 plasmid was constructed using Q5 Site-Directed Mutagenesis Kit (NEB) by introducing the linked missense variants in pAAV-CBh-Flag-WT-SIRT6.

### Lentiviral preparation and transduction

To generate lentiviral particles, HEK293T cells were co-transfected with Plenti4-GFP-progerin, pLV-Neo-EF1A-Flag-VIM or pLV-Neo-EF1A-Flag, along with psPAX2 (Addgene, 12260) and pMD2.G (Addgene, 12259) packaging plasmids, using Lipofectamine 3000 (Thermo Fisher). Viral supernatants were collected 48 hours post-transfection, filtered through a 0.45 uM filter, and concentrated using the Lenti-X Concentrator (Takara) in accordance with the manufacturer’s protocol. Cells were transduced with the concentrated lentivirus in the presence of 10 μg/ml polybrene (Millipore). For GFP-progerin overexpression, cells were sorted using the BD Influx cell sorter after expansion one passages post-transduction to ensure all cells were successfully transduced. For VIM overexpression, transduced cells were selected with 200 ng/ml G418 (Thermo Fisher) to establish stable neomycin-resistant populations for downstream analyses.

### AAV packaging and transduction

To generate crude AAV particles, HEK293T cells were co-transfected with pAAV-CBh-EV (empty vector), pAAV-CBh-Flag-WT-SIRT6 or pAAV-CBh-Flag-CentSIRT6, along with pAAV2/2 (Addgene, 232199) and pAdDeltaF6 (Addgene, 112867), using Lipofectamine 3000 (Thermo Fisher). Cell and medium were collected 72h post-transfection and were frozen (20 mins) and thawed (10 mins) 5 times to efficiently lyse cells and release AAV particles into solution. Benzonase nuclease (Sigma) were added into the solution and incubated at 37°C for 30 mins. The crude AAV suspension was centrifuged at 3,000 × g for 10 minutes to remove residual cell debris and concentrated using an Amicon Ultra-15 Centrifugal Filter Unit (Sigma). The concentrated viral particles were resuspended and added onto HGPS fibroblasts for transduction. HGPS fibroblasts were collected 7 days post AAV transduction.

### RNA-seq library preparation

Total RNA of hMSCs was isolated with the RNeasy Mini Plus Kit (Qiagen) following the manuals and then the integrity of RNA was checked by Bioanalyzer (Agilent). For hMSCs at early passages, RNA-seq libraries were constructed using NEBNext Ultra II RNA library Prep Kit and rRNA Depletion Kit (NEB) according to the manufacture’s protocol and paired-end reads were generated from Illumina NextSeq 550 at the Genomic Core of Albert Einstein College of Medicine. For hMSCs overexpressing GFP-progerin, RNA-seq libraries were constructed using Ribo-Zero Plus rRNA Depletion Kit (Illumina) according to the manufacture’s protocol and paired-end reads were generated from Illumina NovaSeq 6000 at Columbia University Genome Center.

### RNA-seq data processing

Trim Galore (0.6.10) was used to trim adaptor sequences and filter out low-quality reads. Processed reads were aligned to the human reference genome (hg38) using STAR (2.7.11b) and counted by featureCounts from Subread 2.0.2 package at gene level. Differential gene expression was analyzed by DESeq2 and assessed for Gene ontology or Gene Set Enrichment Analysis by clusterProfiler 4.0. TPM expression values were calculated using TPMCalculator. All DEG analysis results generated by DESeq2 were provided in Table. S2-S3.

### Transposable element analysis

To evaluate the expression levels of repetitive elements, the cleaned reads were mapped to the human reference genome (hg38) STAR software (version 2.7.11b) with the parameter --winAnchorMultimapNmax 100 -outFilterMultimapNmax 100 --outFilterMismatchNoverLmax 0.05. TE annotation file GRCh38_GENCODE_rmsk_TE.gtf was obtained from https://www.mghlab.org/software/tetranscripts. Transposable element quantification and the differential analysis were computed using the TEtranscripts software (version 2.2.3). In brief, TEtranscripts simultaneously counted the gene abundances and transposon abundances and utilized DESeq2 for the differential analysis. All differentially expressed transposable element analysis results generated by TEtranscripts were provided in Table. S4-S5.

### Statistical analysis

The statistical analyses were performed using PRISM software (Graphpad Software). Comparisons were performed with unpaired two-tail student’s t-test unless otherwise stated. P < 0.05 was defined as statistically significant.

## Supplementary Material

Supplementary Files

This is a list of supplementary files associated with this preprint. Click to download.

- SupplementaryFiglegends.docx
- Fig.S1S6.docx
- Tables.xlsx

## Data Availability

All sequencing data were deposited to or fetched from Sequence Read Archive (SRA) or Gene Expression Omnibus (GEO) in the National Center for Biotechnology Information. The accession numbers were as follows: RNA-seq of WT and CENT hMSCs at early passage (PRJNA862192), RNA-seq of WT and CENT hMSCs expressing GFP-progerin (PRJNA1272066), RNA-seq of proliferating and senescent hMSCs (PRJNA1272555), RNA-seq of *SIRT6*-knockout hMSCs (GSE64642).
